# The leukoaraiosis is more prevalent in the large artery atherosclerosis stroke subtype among Korean patients with ischemic stroke

**DOI:** 10.1186/1471-2377-8-31

**Published:** 2008-08-07

**Authors:** Seung-Jae Lee, Joong-Seok Kim, Kwang-Soo Lee, Jae-Young An, Woojun Kim, Yeong-In Kim, Bum-Soo Kim, So-Lyung Jung

**Affiliations:** 1 Department of Neurology, The Catholic University of Korea, Seoul, South Korea; 2 Department of Neurology, National Cancer Center, Koyang-city, Kyunggi-province, South Korea; 3 Department of Radiology, The Catholic University of Korea, Seoul, South Korea

## Abstract

**Background:**

Several studies have suggested that the specific stroke subtype may influence the presence of leukoaraiosis in patients with ischemic stroke. We investigated the association between stroke subtype and leukoaraiosis in Korean patients with ischemic stroke by MRI.

**Methods:**

There were 594 patients included in this study that were classified as large artery disease, lacune and cardioembolic stroke. For large-artery disease, the analysis focused on the intracranial or extracranial location of the stenosis, and the multiplicity of the stenotic lesions. Leukoaraiosis grading was performed according to the Atherosclerosis Risk in Communities Study.

**Results:**

There was a significant association between leukoaraiosis and the stroke subtypes; the large-artery-disease group had a higher prevalence of leukoaraiosis than did the other groups (55.4% in the large-artery-disease group, 30.3% in the lacunar group and 14.3% in the cardioembolic group, P = 0.016 by chi-square test). On the multivariate linear regression analysis, age, the presence of hypertension, previous stroke and stroke subtype were independently associated with the presence of leukoaraiosis. In the sub analysis of the large-artery-disease group, the leukoaraiosis had a tendency to be more prevalent in the mixed and intracranial stenosis group than did the extracranial stenosis group (45.5% in the mixed group, 40.3% in the intracranial group and 26.9% in the extracranial group, P = 0.08 by chi-square test).

**Conclusion:**

The association of leukoaraiosis with large-artery disease in this study might be due to the relatively high prevalence of intracranial occlusive lesions in Korean stroke patients compared to other ethnic groups.

## Background

The term leukoaraiosis (LA) refers to lesions of altered signal intensity on computed tomography (CT) and magnetic resonance imaging (MRI) in the periventricular and subcortical white matter. LA is found during the normal aging process, and in the patients with cerebrovascular disease. It also constitutes the core pathology of Binswanger's disease, a type of vascular dementia. The association of LA with lacunar infarcts rather than territorial infarcts is well documented [1-7]. However, most prior studies have been based on CT findings, not MRI, and reported from western countries.

In the present study, we analyzed the association between stroke subtype and LA in Korean stroke patients using MRI.

## Methods

### 1. Patients

We initially included 963 consecutive acute ischemic stroke patients admitted to the neurology department from July 2003 to June 2007. All patients underwent detailed clinical evaluation including laboratory tests, chest radiography, transcranial Doppler study, electrocardiography and 24 hour Holter monitoring. In addition, transthoracic echocardiography and brain magnetic resonance imaging (MRI), contrast-enhanced MR angiography (MRA) and/or cerebral angiography were obtained. All results from the evaluations were analyzed according to the diagnostic criteria for stroke mechanisms and etiology based on the TOAST subtype classification system [8]. Among the initial patients, 369 were categorized into stroke of undetermined etiology (mean age ± SD, 68.1 ± 10.2; age range, 31–87 years) and were excluded from the study: of those patients, 245 patients were classified as stroke of two or more potential etiology (164 with lacune plus large-artery disease, 54 with large-artery disease plus cardioemobolism and 27 with lacune plus cardioembolism), 21, 76 and 27 patients were classified as groups of negative evaluation, incomplete evaluation and other determined etiology, respectively.

Finally, 594 cases with large artery disease (297 patients), lacune (193 patients) and cardioembolic stroke (104 patients) were enrolled in this study. The ethics committee at our institution approved the study protocol, and all subjects provided written informed consent.

### 2. Risk factor evaluation

The clinical information included age, gender, history of hypertension (defined by the use of an antihypertensive agent before admission or a systolic pressure > 140 mmHg or diastolic pressure > 90 mmHg demonstrated on repeated examinations at least one month after presentation with a stroke), diabetes mellitus (defined as a fasting blood glucose level > 126 mg/dl or a history of being treated for diabetes mellitus) and hyperlipidemia (defined as a total cholesterol level > 200 mg/dl or a low-density lipoprotein cholesterol > 130 mg/dl at the time of presentation or a history of treatment). In addition, regular cigarette smoking, a previous history of ischemic stroke and heart disease (defined as a known history or clinical demonstration of any heart disease, including myocardial infarction, angina pectoris, congestive heart failure, or arrhythmia) were noted

### 3. MR imaging and LA grading

All patients enrolled underwent conventional MRI on a 1.5-T system (Signa 1.5-T TwinSpeed, General Electric Medical Systems and Archieva 1.5-T, Philips Electronics) within 7 days of the stroke onset. The conventional MRI consisted of transverse T2/T1-weighted, fluid-attenuated inversion recovery (FLAIR) sequences and sagittal T1 with 5-mm-thick slices. Diffusion-weighted imaging was obtained in the transeverse plane using a single-shot echoplanar, spin-echo pulse sequence. A three-dimensional time of flight MRA of the intracranial arteries and contrast-enhanced MRA of the head and neck were also performed on the same system using a neurovascular coil.

LA was defined as a periventricular white matter lesion with hyperintensity on T2- weighted and FLAIR images and without prominent hypointensity on T1-weighted images. The LA grading was according to the Atherosclerosis Risk in Communities (ARIC) study [9,10]. Three trained neurologists and two neuroradiologists, blinded to patient data and stroke subtype, graded the LA by consensus. When evaluating the WMH, new (high signal on diffusion-weighted image) and old (definitely low signal on T1-weighted image) infarcts were excluded. If one or both sides of the brain were focally abnormal, estimates were based on the uninvolved side with the principle of symmetry assumed.

### 4. Patterns of stenotic lesions in large artery disease

The evaluation of the arterial stenosis was performed by the investigators and > 50% of signal loss on the MRA was considered to be a "significant stenosis" for the classification of stroke subtype and the categorization of stenosis pattern. The locations of significant stenosis were categorized as located in the intracranial or extracranial arteries. For the internal carotid artery, an intracranial location was defined when the stenotic lesion was distal to the ophthalmic artery. For the vertebral artery, the differentiation was made at the point where the artery pierced the dura at the level of the foramen magnum. The intracranial extent of the stenosis included up to the M2 of the middle cerebral artery (insular segment which terminates at the circular sulcus of the insula before the operculum) and the A2 segment of the anterior cerebral artery (ascending segment with inferior forward convexity) in the anterior circulation, and the P2 segment of the posterior cerebral artery (ambient segment which extends from the junction between the posterior communicating artery and the posterior cerebral artery to the posterior aspect of midbrain). The area of the stenotic lesion was divided into intracranial or extracranial and anterior or posterior circulation. The stenoses were described as single or multiple according to the number of the areas involved with the arterial stenosis.

According to the distribution and pattern of the stenotic lesions, the patients with large-artery disease were categorized as intracranial, extracranial and a mixed (intracranial plus extracranial) group, and as multiple- or single-stenosis groups.

### 5. Statistical analysis

We classified persons with leukoaraiosis of grade 3 or higher as having "LA" and of grade 2 or lower as having "little or no LA" [10]. The presence or absence of LA was compared between the patients with risk factors and the patients without, the stroke subtypes, the multiple stenosis and single stenosis groups, and among intracranial, mixed and extracranial stenosis groups by chi-square test or independent t-test. In addition, we compared the severity of LA between the groups of different stroke subtypes by one-way ANOVA test and LSD multiple comparison test. Multiple linear regression analysis was used to determine the factors considered independently associated with leukoaraiosis. Values of P < 0.05 were considered statistically significant.

## Results

Of the 594 patients, 342 (57.6%) were men and 252 (42.4%) were women (mean age ± SD, 66.8 ± 12.1; age range, 27–97 years). In study population, distinct white matter changes were present in 307 patients (51.7%). The association of the presence of LA to age was statistically significant; the LA group had higher age at onset of stroke than the "little or no LA" group. The LA was more frequently observed in female gender, the patients with hypertension and a history of previous ischemic stroke than in the patients without this history (P < 0.05). There was no significant association between the LA and diabetes mellitus or hyperlipidemia; a negative correlation was found with the smoking status and the presence of heart disease (table 1).

**Table 1:** Characteristics of stroke patients by leukoaraiosis.

	Leukoaraiosis			
Characteristics	Present (n = 307)	Absent (n = 287)	P value	
Age (yr)	72.1 ± 9.8	61.1 ± 11.9	< 0.001	
Gender			0.001	
Male (n = 342)	157 (45.9)	185 (54.1)		
Female (n = 252)	150 (59.5)	102 (40.5)		
Hypertension (n = 383)	229 (59.8)	154 (40.2)	< 0.001	
Diabetes mellitus (n = 236)	133 (56.4)	103 (43.6)	0.390	
Hyperlipidemia (n = 193)	95 (49.2)	98 (50.8)	0.228	
Heart disease (n = 105)	44 (41.9)	61 (58.1)	0.018	
Atrial fibrillation (n = 78)	34 (43.6)	44(56.4)	0.125	
Regular cigarette smoking (n = 153)	57 (37.3)	96 (62.7)	< 0.001	
Previous ischemic stroke (n = 87)	58 (66.7)	29 (33.3)	0.002	
Stroke subtype			0.016	
Large artery disease(n = 297)	170 (57.2)	127 (42.8)	0.031^a^	0.006^b^
Lacunar(n = 193)	93 (48.2)	100 (51.8)	0.198^b^	
Cardioembolic (n = 104)	44 (42.3)	60 (57.7)		

Values represent mean ± SD and number of patients with percentages in parenthesis

The two groups were compared by two-sample *t-*test for continuous variables and chi-square test for nominal variables.

^a ^Comparison is with lacunar group, based on the Chi-square test.

^b ^Comparison is with cardioembolic group

Among the 307 patients with LA, 170 patients (55.4%) were in the large-artery-disease group, 93 patients (30.3%) in the lacunar group and 44 patients (14.3%) in the cardioembolic group (P = 0.016 by chi-square test, table 1). In addition, there was overall a significant association between the LA grade and the stroke subtype: the large-artery-disease group had more severe LA disease than did the other groups. Although the lacunar group tended to have a higher LA grade than did the cardioembolic group, there was no significant difference in the LA grade in comparisons between the two groups (table 2). On the multivariate linear regression analysis, using the variables (age, gender, the presence of hypertension, diabetes mellitus, hyperlipidemia, smoking, previous stroke, and stroke subtype), age, the presence of hypertension, previous stroke and stroke subtype were independently associated with the presence of LA (Table 3).

**Table 2:** Leukoaraiosis grade by stroke subtype.

	Stroke subtype			
	Large artery disease	Lacunar	Cardioembolic	P value
	(n = 297)	(n = 193)	(n = 104)	
LA grade (mean ± SD)	3.37 ± 1.98	2.86 ± 1.95	2.54 ± 1.71	< 0.001
LSD, P value	0.004^a^, < 0.001^b^	0.170^b^		

P value refers to the overall association between leukoaraiosis grade and stroke subtype, computed from one-way ANOVA test.

^a ^Comparison is with the lacunar group, based on LSD multiple comparison tests.

^b ^Comparison is with the cardioembolic group

**Table 3:** Multiple linear regression analysis for the relationship between leukoaraiosis and potential confounding variables.

	*t*	*p*
Age	11.130	< 0.001
Gender	1.056	0.292
Hypertension	4.132	< 0.001
Diabetes mellitus	0.611	0.541
Hyperlipidemia	- 0.117	0.907
Smoking	0.550	0.583
Previous stroke	2.440	0.015
Stroke subtype	2.449	0.013

Data are t (p value) of the correlation.

^a^R^2 ^= 0.503

In the large-artery-disease group, there was a borderline association between the LA and the stenotic areas; the LA was most prevalent in the mixed stenotic group, next was the intracranial stenosis group and the extracranial stenosis group was the least affected. In addition, a more LA was observed in patients with multiple arterial stenoses than in patients with single arterial stenosis (table 4).

**Table 4:** Association between the pattern of stenosis and leukoaraiosis in large-artery-disease group.

	Leukoaraiosis			
			
	Present (n = 170)	Absent (n = 127)	P value	
Distribution of stenosis			0.080	
Mixed (n = 101)	46 (45.5)	55 (54.5)	0.245^a^	0.023^b^
Intracranial (n = 144)	58 (40.3)	86 (59.7)	0.070^b^	
Extracranial (n = 52)	14 (26.9)	38 (73.1)		
Number of stenotic lesions			0.019	
Multiple (n = 155)	71 (45.8)	84 (54.2)		
Single (n = 142)	47 (33.1)	95 (66.9)		

Values represent number of patients with percentages in parenthesis

The two groups were compared by chi-square test.

^a ^Comparison is with intracranial group

^b ^Comparison is with extracranial group

## Discussion

Although the pathophysiology of LA remains speculative, there is evidence to suggest that LA may be linked to cerebral ischemia [11-24]. Selective injury to the cerebral white matter has been noted in a limited number of human conditions characterized by hypoxia/ischemia of the brain such as carbon monoxide poisoning and therapeutic occlusion of the internal carotid artery [11-14]. It has been assumed that the ischemic insult, responsible for LA, results from the vulnerable nature of the long penetrating end-arteries that feed the deep white matter [15,16]. LA has been associated with increasing age, arterial hypertension and other cerebrovascular risk factors [17-22]. In addition, white matter lesions similar to LA can be induced in rats or gerbils by ligating the bilateral common carotid arteries [23,24].

Although the previous studies of risk factors for LA have shown different results, advanced age and hypertension have been consistently reported to be highly associated with LA [1]. One recent study indicated that diabetes mellitus seems to be a risk factor for progression rather than new LA development [7]. In our study, similar to previous studies in other ethnic groups, LA was associated with age, hypertension and previous history of ischemic stroke. However, it had no relation with hyperlipidemia and diabetes mellitus. In addition, there was a negative correlation between regular cigarette smoking and LA, as previously reported in one study [21]. However, the relationship between smoking and brain disorder is controversial, and the negative association with LA shown in this study should be discussed in further future study. These differences between the results of previous researches may be due to study variations in patients and/or the definitions of LA used by different investigators.

The LA was more frequently observed in the large-artery-disease group than the other subtypes. In addition, the large-artery-disease group had more severe LA than did the other group. The cardioembolic group had the lowest prevalence of LA although there was no significant difference when compared to the lacunar group. This finding suggests that the hypoperfusion that results from large-artery occlusion might be more important to the progression and aggravation of LA. This is supported by the fact that the periventricular white matter, vulnerable to LA, was the distal irrigation field or border zone; these areas are prone to ischemia under conditions of moderate blood flow deficits.

However, the results of our study are in general not consistent with prior reports. In most previously reported studies, LA was strongly associated with lacunar strokes rather than non-lacunar, territorial strokes [1-7]. In addition, an inverse correlation between high-grade (> 50%) stenoses of the extracranial arteries and white matter changes has been reported [1,21,25-27]. These observations have been explained by the hypothesis that reduced blood perfusion, in the patients with high-grade stenoses of the extracranial carotid arteries, might alleviate damage to intracerebral arteries by decreasing tensile stress on the arterial walls [21,28]. However, these studies did not divide the territorial infarcts into large artery disease or cardioembolic strokes. Moreover, most prior studies were from western countries, and the results were based on CT findings not MRI, which is better for assessing white matter lesions.

In the present study, most of the patients in the large-artery-disease group had intracranial stenotic lesions (82.5%); extracranial lesions were uncommon. The LA tended to be more frequent in patients with intracranial stenoses than in the patients with extracranial stenoses alone. The intracranial location for cerebrovascular atherosclerosis is characteristic of strokes in the Asian population [29-31]. Therefore, the more prevalent LA in patients with large-artery-disease compared to the other subtypes in this study might be explained by the high prevalence of intracranial stenoses in Korean stroke patients.

Unlike extracranial stenoses, more prevalent in Caucasians, intracranial stenoses may have a different pathogenesis contributing to the development and progression of LA. First, atherosclerotic stenoses of intracranial arteries can directly occlude the orifice of numerous small perforators penetrating into the deep brain parenchyma. The occlusion of the small perforators promotes extensive hypoperfusion of the periventricular region that is vulnerable to ischemia. Second, the periventricular border zone, under ischemic conditions induced by the stenoses of intracranial arteries, might have less opportunity to be compensated by blood flow via major collateral channels such as the anterior or posterior communicating artery, which can be recruited without difficulty in stroke patients with stenosis of extracranial vessels. Third, compared with the emboli from the stenotic lesions of extracranial arteries, those from an intracranial location might not easily be cleared away by travel along with the blood flow, probably because of the close proximity to the perfused brain tissues.

The limitations of this study include the following. One methodological problem is that our results are based on a cross-sectional sample. The longitudinal effect of large-artery atherosclerosis on periventricular white matter changes could not be accurately assessed in this study. In addition, a selection bias might have been present because 369 (38.3%) of the 963 patients were excluded due to the diagnosis of a stroke of undetermined etiology or other determined etiology; we used very strict criteria for the classification of subjects into specific stoke subtypes. For example, suspected large artery disease with < 50% signal loss of the proximal artery on MRA, or supratentorial subcortical infarcts of < 1.5 cm with > 50% stenosis of the parent artery were classified as strokes of undetermined origin. The application of this criterion would increase the specificity and lessen the likelihood of misclassification of patients in the other categories [8]. Moreover, the quantitative analysis of LA by a 0-to-9 grading system might not reflect an accurate estimation of the LA severity. Additional grading systems including volumetric methods for LA are needed.

## Conclusion

The results of this study showed that LA is significantly associated with large-artery-disease rather than other stroke subtypes including small vessel disease in Korean stroke patients. The differences between our study and previous reports might be due to the high prevalence of intracranial occlusive lesions in patients with cerebrovascular atherosclerosis in the Asian population. Different from the high-grade stenoses of extracranial arteries, the stenoses of intracranial arteries might induce extensive white matter changes by interrupting blood flow to the periventricular border zone vulnerable to ischemia.

## Competing interests

The authors declare that they have no competing interests.

## Authors' contributions

SJL and JSK conceived and coordinated the study, analysed the data and drafted the initial manuscript. All authors were involved in initial literature search and collection of data. Review of initial manuscript for major intellectual content was done by SJL and JSK. All authors read and approved the final manuscript.

## Pre-publication history

The pre-publication history for this paper can be accessed here:


